# Treatment of alopecia areata with Diphenylcyclopropenone: methodology based on the principles of allergic contact dermatitis^☆^

**DOI:** 10.1016/j.abd.2020.08.036

**Published:** 2021-12-06

**Authors:** Andressa Sato de Aquino Lopes, Rosana Lazzarini

**Affiliations:** Medical Department of Clínica de Dermatologia, Irmandade da Santa Casa de Misericórdia de São Paulo, São Paulo, SP, Brazil

Dear Editor,

Diphenylcyclopropenone (DPCP) is a chemical substance that induces a cellular immune response and, therefore, allergic contact dermatitis (ACD). Its action is based on the concept of antigenic competition, inducing the formation of TCD8 lymphocytes, which inhibit the active perifollicular immune response, allowing hair growth.^1^

DPCP is a therapeutic option for alopecia areata (AA), especially in extensive cases, with a variable response, but repilation rates in more than 50% of cases.^1^ Side effects are common, sometimes severe, such as acute eczematous reactions, in addition to lymphadenopathy, pruritus, hyperpigmentation, and flu-like symptoms, among others.^2^

Drug utilization varies, lacking methodological standardization.^2^, ^3^, ^4^ This service uses a methodology based on the principles of ACD. This case report aims to demonstrate the steps of DPCP use in AA. This standardization allowed comparing data and reducing side effects due to drug inappropriate use.

The product is purchased at 2% in acetone and stored in the refrigerator in a dark bottle. The dilutions are prepared during the appointments and applied weekly with moistened flexible swabs.

A 44-year-old male with universal AA was sensitized with 2% DPCP on 2 × 2 cm filter paper on the back for 48 hours, inducing the ACD induction phase. After 2 weeks, he was submitted to a patch test with DPCP, at 0.1%; 0.05%, and 0.02% concentrations, with readings after 48 and 96 hours, and responses 3+, 2+ and 2+ (Fig. 1) respectively, according to the previously established standardization. The 0.02% concentration was used on the scalp, where it remained covered and unwashed for 48 hours. The concentration was increased weekly to 0.1% when moderate pruritus and erythema were obtained. Repilation was acceptable after 24 weeks, without severe reactions (Fig. 2).

**Figure 1:**
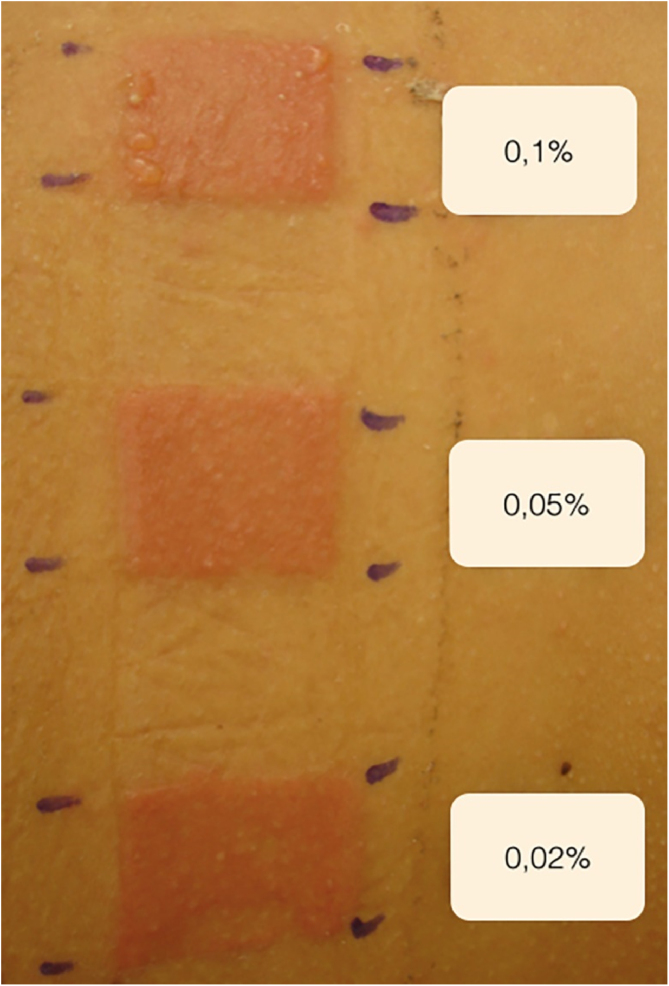
Reading of the patch test after 72 hours.

**Figure 2:**
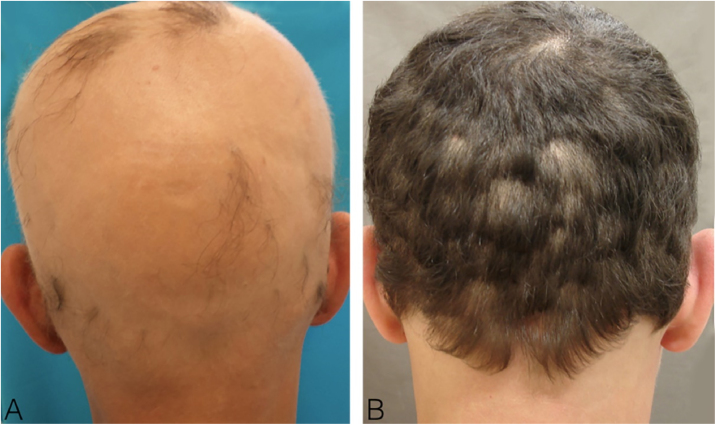
(A), At start of treatment; (B), after 24 weeks of DPCP treatment.

The patch test assesses whether there was sensitization to the product and predicts the best concentration with which to start treatment, choosing the one with the lowest positivity, minimizing adverse effects. If the responses are intense, the concentrations are reduced, and sometimes the applications are spaced out at intervals of two to four weeks. The concentrations depend on the response of each individual.

Therapeutic failure, that is, the absence of repilation, is considered after 180 days of regular application. If there is a response, the applications are maintained until the best possible effect is attained (up to one year).

DPCP is a drug that provides good response rates in severe cases; however, the protocols are not yet standardized. Due to common and sometimes severe side effects, strict monitoring of sensitization and control of the concentrations are necessary throughout the treatment. Moreover, no industry manufactures DPCP in accordance with regulatory standards for drug development.^5^ In the present environment, there is no regulation for its use, although it has been part of the therapeutic arsenal for AA treatment for many years.

## Financial support

None declared.

## Authors’ contributions

Andressa Sato de Aquino Lopes: Approval of the final version of the manuscript; design and planning of the study; drafting and editing of the manuscript; critical review of the literature; critical review of the manuscript.

Rosana Lazzarini: Approval of the final version of the manuscript; drafting and editing of the manuscript; intellectual participation in the propaedeutic and/or therapeutic conduct of the studied case; effective participation in research orientation; critical review of the manuscript.

## Conflicts of interest

None declared.
